# Estimation of Digital Porosity of Electrospun Veils by Image Analysis

**DOI:** 10.3390/polym16020300

**Published:** 2024-01-22

**Authors:** Guadalupe Cuahuizo-Huitzil, Octavio Olivares-Xometl, Paulina Arellanes-Lozada, José Oscar Laguna Cortés, Janette Arriola Morales, Claudia Santacruz-Vázquez, Verónica Santacruz-Vázquez

**Affiliations:** 1 Facultad de Ingeniería Química, Benemérita Universidad Autónoma de Puebla, Av. San Claudio y 18 Sur, Puebla 72570, Mexico; guadalupe.cuahuizo@alumno.buap.mx (G.C.-H.); octavio.olivares@correo.buap.mx (O.O.-X.); paulina.arellanes@correo.buap.mx (P.A.-L.); janette.arriola@correo.buap.mx (J.A.M.); 2 Departamento de Ciencias Básicas, Tecnológico Nacional de México—Instituto Tecnológico de Puebla, Av. Tecnológico 420, Puebla 72220, Mexico; jose.laguna@puebla.tecnm.mx

**Keywords:** nanofibers, SEM, digital porosity, emulsions, poly(vinyl alcohol)

## Abstract

The present work reports on an empirical mathematical expression for predicting the digital porosity (DP) of electrospun nanofiber veils, employing emulsions of poly(vinyl alcohol) (PVOH) and olive and orange oils. The electrospun nanofibers were analyzed by scanning electron microscopy (SEM), observing orientation and digital porosity (DP) in the electrospun veils. To determine the DP of the veils, the SEM micrographs were transformed into a binary system, and then the threshold was established, and the nanofiber solid surfaces were emphasized. The relationship between the experimental results and those obtained with the empirical mathematical expression displayed a correlation coefficient (R^2^) of 0.97 by employing threshold II. The mathematical expression took into account experimental variables such as the nanofiber humidity and emulsion conductivity prior to electrospinning, in addition to the corresponding operation conditions. The results produced with the proposed expression showed that the prediction of the DP of the electrospun veils was feasible with the considered thresholds.

## 1. Introduction

Among the different techniques that have been employed to obtain fibrous membranes from synthetic or natural polymers for the development of materials that can be applied in the food, pharmaceutical and biomedical industries, among others, electrospinning has played a major role [1,2,3]. Membranes for their industrial use, synthesized from micro- and nano- electrospun fibers, have offered advantages due to the fact that their surface area per unit of volume and porosity can vary. Furthermore, these features are a function of the properties of the solution or emulsion to be electrospun: concentration, density, viscosity, conductivity and surface tension [4]. Additionally, electrospinning conditions such as flow rate, voltage, temperature and injector-collector distance have to be taken into account [5]. As for the characterization of porous materials, conventional techniques like mercury intrusion porosimetry (MIP), X-ray diffraction, centrifugal porosimetry and nitrogen sorption porosimetry (NSP), among others, are based on physical methods that try to represent the totality of the sample [6,7,8]. With the use of the MIP and NSP techniques, it is very likely that the membrane be destroyed at high pressures, for the pores are not rigid enough, given the characteristics of the electrospun veils [9].

Digital image analysis (DIA) has been gaining importance in the study of porous materials. Different authors have discussed the importance of employing image analysis techniques by means of SEM micrographs to estimate the porosity of cellulose-based foams and aerogels [10] and in ceramic and organic materials, among others [11]. For these reasons, DIA is considered as an alternative method for estimating porosity by seizing micro- and macropores present in materials.

Wu et al. [12], for characterizing quantitatively the morphology of membranes through SEM DIA, defined parameters such as geometrical distribution of pore size, surface porosity and fractal dimension of membrane pores; the latter reflects the irregularity degree of the membrane pores. Likewise, the obtained results contributed to understanding the membrane morphology and pore formation mechanisms. Crawford et al. [13] constructed a Java plugin codified for the open code program ImageJ intended for the automated analysis of alluvial mineral and tracer images to record morphological parameters like area, perimeter and Fourier analysis parameters. The results of studying gold morphological changes during alluvial transport showed that the data produced by this model defined a quantitative relationship between the distance and transport form. Grove et al. [14] determined the total optical porosity of thin sections impregnated with blue resin by employing a macro jPOR file for ImageJ. The results were compared with the point counting method; however, jPOR provided results that were comparable to those obtained by point counting, which requires more time. Haeri et al. [15] employed the Java-based-open-code software ImageJ 1.51j8 developed by the National Institutes of Health to calculate the mean size and distance between particles and concluded that this software is particularly useful in the analysis of synthetic and natural porous constructions known as scaffolds that are usually used in the field of regenerative medicine and tissue engineering. The architecture of these scaffolds, including the size and pore density, affected significantly their interaction with biological cells and scaffold mechanical properties. Pal et al. [16], by means of representative rock fragments, obtained images of rock cores by SEM. These images were processed and analyzed with the software ImageJ to produce 2D and 3D porosity. The porosity data by the DIA technique were compared with the porosity given by using a helium gas porosimeter, finding that the 2D porosity is between 14.543–45.328% for carbonates and 3.895–35.561% for sandstone, whereas 3D porosity is between 7.8–9892% for carbonates and 3.52–9.75% for sandstone. The estimated values of 2D porosity fell within the expected interval, whereas the 3D porosity values were underestimated in comparison with the employed technique; for this reason, it was concluded that this technique is useful for establishing 2D porosity. Daraei et al. [17] analyzed the microstructure of blood clots, because the fiber diameter and clot porosity can be altered by medicaments or cardiovascular disease. They used the ImageJ complement called DiamterJ and analyzed SEM images of fibrin meshes, reporting the diameter measurements and porosity and comparing them with manual measurements and concluded that the algorithms resulted suitable for establishing the diameter through image analysis; the measurements were adjusted to the clot biophysical characteristics and manually determined values. Tan et al. [18] estimated the porosity of natural rocks by means of SEM data and a pore and crack analysis system. These authors concluded that the digital analysis was capable of identifying accurately the pore size and porosity values, which agreed with experimental data. The relationship between the bidimensional porosity estimated from digital analysis and the tridimensional porosity obtained from laboratory experiments was established.

In many applications of nanofiber membranes as filters and scaffolds for tissue engineering, it is important to know the porosity of several layers, because the materials not only have high specific surface area, but also provide an inductive structure for tissue engineering. Understanding the dynamic effects of the tridimensional matrix structure and pore size in the veils is the first step that requires the optimal design of materials for tissue or membrane engineering.

In the literature, a reduced number of mathematical models capable of describing a specific phenomenon or of predicting the characteristics of electrospun nanofibers is reported. This fact is due to the fact that the electrospinning process is complex and involves electrostatic processes of momentum and mass transfer. In this sense, Fridrikh et al. [19] presented equations for predicting the formation of nanofibers as a function of material properties such as conductivity (k), electric permittivity (ε), dynamic viscosity (μ), surface tension (γ) and density (ρ), and also of operative characteristics like flow rate (Q), applied electric filed (𝐸∞) and electric current (I). Conversely, Stepanyan et al., in 2014 [20] and 2016 [21], based their studies on the nanofiber elongation kinetics and determined the nanofiber radio in a single injector. Maurya et al. [22] proposed a predictive model for the fiber diameter considering the application of artificial neural networks, due to the existence of nonlinear relationships between the process variables, and poly(vinyl alcohol) and ferrous compounds. However, mathematical models for predicting the porosity of electrospun membranes are not reported in the literature. 

As a result, it can be concluded that a micrometric morphological study of materials can enable researchers to approach and deepen the knowledge of transport phenomena and diffusion mechanisms associated with the mass transfer of porous materials. Parameters such as diameter and pore size can be determined quantitatively through the analysis of images, which allows the characterization of the microstructure and complexity of materials.

With the help of image analysis, the present study proposes the possibility of measuring the DP of various veil surface layers of experimentally electrospun nanofibers as a function of the main parameters of the electrospinning process, such as nanofiber moisture and electrospinning time, which are considered as critical experimental parameters in the mathematical expression employed to determine the DP of veils. The electrospun nanofiber veils were synthesized by employing PVOH emulsions due to their amphiphilic, emulsifying and encapsulating properties [23,24]. Furthermore, since PVOH possesses a polar structure, because of the presence of the OH^-^ group, it gives to it surfactant properties that are useful in the formation of emulsions with hydrophobic compounds [25,26].

## 2. Materials and Methods

### 2.1. Materials

PVOH crystals (Sigma-Aldrich, St. Louis, MO, USA) were dissolved in distilled water to form a PVOH solution at 10% *w*/*w*. Commercial olive oil (OO) and orange essential oil (OEO) were incorporated into the PVOH solution by employing a magnetic stirrer for 1 h at 50 ± 1 °C and 600 rpm until producing homogeneous PVOH-OO and PVOH-OEO emulsions.

### 2.2. Characterization of Emulsions

The electric conductivity of the PVOH-OO and PVOH-OEO emulsions was determined by means of a piece of equipment Conductronic model PC18 at 25 ± 1 °C with measurements that were done in triplicate. The viscosity measurements of the mentioned emulsions were carried out using a rheometer Anton Paar model RheolabQC, (Anton Paar, Ashland, VA, USA) with the concentric cylinder configuration DG24 at 25 ± 1 °C and employing the software Star Rheoplus 3.0x [4].

### 2.3. Electrospinning and Characterization of Electrospun Veils

The PVOH-OO and PVOH-OEO emulsions were electrospun in a piece of equipment electrospinning horizontal Prendo, Espin 50 kV, at ambient temperature to produce nanofibers and form veils. The measurement of the initial moisture of the electrospun veils was carried out by the oven drying method. The veils measured 7 cm wide × 7 cm long and were removed from aluminum paper and placed in an aluminum tray. The samples were dehydrated in triplicate at 70 ± 1 °C for 24 h. The SEM micrographs of the PVOH-OO and PVOH-OEO emulsions were analyzed by means of a microscope model JSM-6610LV with a magnification of 5000×. The digitization of the SEM micrographs was analyzed with the image processing software ImageJ v1.51j8 to determine the DP, whereas, for the orientation of nanofibers, the Orientation J algorithm was used. This algorithm analyzed the SEM images of the nanofibers in a binary format of 8 bits to establish the orientation of the nanofibers present in the input image. Furthermore, histograms were produced to indicate the number of structures in a given direction. 

### 2.4. Determination of the DP of Electrospun Veils

The original SEM images of the electrospun veils were transformed by employing a gray scale; the scale was established as measuring unit, and then the gray-scale images were converted into 8-bit binary images (black and white). A filter to reduce noise and a thresholding process based on the grouping or reduction of a gray level to a binary image were used. Once the threshold was set, the segmentation of the measurable regions was obtained from the binary images, where the black color represented the porous region and the white one corresponded to the solid region [27].

**Figure 1:**
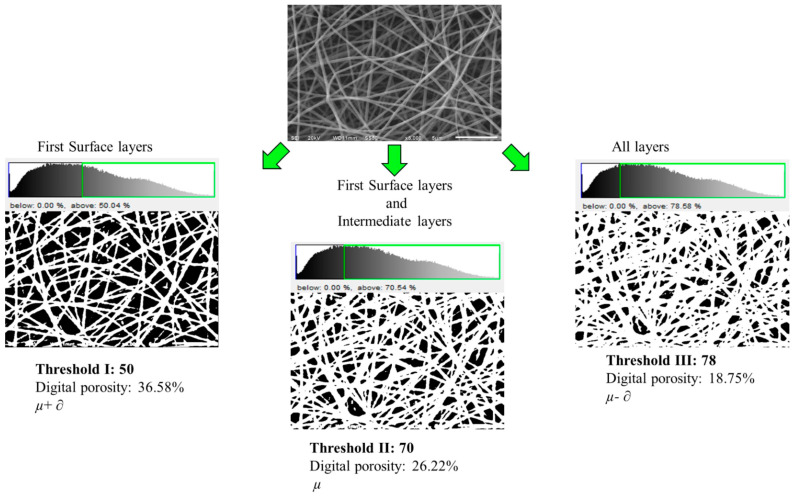
Micrograph of PVOH 10% *w*/*w* with different thresholds.

Figure 1 shows the image analysis of the PVOH 10% *w*/*w* sample with different thresholds, determined as functions of the mean (*µ*) and standard deviation (∂) of the pixel values of the SEM micrographs, surface layers (*µ* + ∂), surface and intermediate layers (*µ*) and all the layers (*µ* − ∂)^2^. From the analysis of the images, three thresholds were found to transform the original image into binary form. The characterization of the thresholds was carried out as a function of the image saturation with the following thresholds: threshold I (0–50%), threshold II (0–70%) and threshold III (0–78%). It was shown that the DP analysis is a function of the image threshold, where, by changing the threshold, various nanofiber layers are observed. The reflection of the upper layers of the nanofiber images is higher than that of the lower layers, then, the intensity of the pixels in the nanofiber upper layers is higher than that of the inferior layers. As observed in Figure 1, with threshold I, only the veil surface layers are observed, thus reporting higher DP, whereas with threshold II, the intermediate layers are joined to the uppermost surface layers, which gave DP values that were lower than those of threshold I. Finally, with threshold III, all the veil visible layers are displayed, thus yielding lower DP as a result of the layer overlapping.

Porosity is the ratio of empty volume (pores) to the system total volume [7], as indicated in Equation (1):

(1) $$\phi =\frac{Vp}{V_{T}}\times 100$$

where ϕ is the porosity, $V_{P}$ is the volume of pores and $V_{T}$ is the total volume. In the present study, the DIA technique was employed [28] to determine the DP, which consisted of identifying the pores in the studied images to calculate afterward the occupied total surface with respect to the total area. This way, the abundance of pores or DP was established as a percentage (%A), as shown in Equation (2):

(2) $$\phi_{D}=\%A=\frac{Ap\;}{A_{T}}\times 100$$

where $\phi_{D}$ is the abundance of pores or DP, $A_{P}$ is the area of pores and $A_{T}$ is the total area. 

### 2.5. Prediction Model and Error Analysis

The prediction model of preceded DP (${DP}_{P})$ was established through a fitting method, employing operation (voltage, injector-collector distance and electric current) and solution (conductivity) parameters of the electrospinning process; the initial and final moisture contents of the electrospun veils and electrospinning time were also taken into account.

The DP experimental value $\left(\phi_{Dexp}\right)$ of the electrospun samples was used to validate the prediction model of (${DP}_{P})$. In addition, in order to know the quality of the fitting, the percentage error (*Pe*), correlation coefficient (R^2^), residual sum of squares (RSS) and mean squared error (MSE) were calculated according to Equations (3)–(6), severally:

(3) $$Pe=\frac{\left|\phi_{Dexp}-\phi_{Dpred}\right|}{\phi_{Dexp}}\times 100\%$$

(4) $$R^{2}=1-\frac{\sum_{i=1}^{N}{\left(\phi_{Dexp}-\phi_{Dpred}\right)}^{2}}{\sum_{i=1}^{N}{{(\phi }_{Dexp}-\phi_{Dm})}^{2}}$$

(5) $$RSS=\sum_{i=1}^{N}({\phi_{Dexp}-\phi_{Dpred})}^{2}$$

(6) $$MSE=\sqrt{\frac{1}{N}\sum_{i=1}^{N}({\phi_{Dexp}-\phi_{Dpred})}^{2}}$$

where $\phi_{Dexp}$ is the DP measured with the image analysis software, $\phi_{Dpred}$ is the ${DP}_{P}$ values of the electrospun veils and $\phi_{Dm}$ is the mean of the experimental values. 

## 3. Results and Discussion

### 3.1. Physical Properties of the Nanofibers

The physical properties of the PVOH-OO and PVOH-OEO emulsions were established prior the electrospinning process, and the results are shown in Table 1. It can be observed that the aqueous emulsions presented high electrical conductivity values, which are related to PVOH. These values fell within the interval ranging from 0.43 to 0.55 mS cm^−1^, and it was found that they depended on the vegetal oil concentration, where the higher oil concentration in the sample, the lower the conductivity. For OO, this phenomenon is associated with the alkyl chain in its structure and its hydrophobic character. As for OEO, the electrical conductivity values were due to benzene functional groups, double bonds and chemical composition of molecules such as d-limonene, α-himachalene, trans-verbenol, linalool, eugenol, acetyl isoeugenol and methyl chavicol, which are, in general, molecules with low hydrophilic capacity [29,30].

The use of the PVOH solution at 10% *w*/*w* allowed the formation of a more defined veil with thicker and homogeneous morphology with nanofiber diameters between 290–307 nm. Unlike the PVOH emulsion at 8% *w*/*w*, the nanofibers presented diameters between 173 and 179 nm. The reported diameters are close to those obtained by Rošic et al. [31] for nanofibers electrospun with PVA at 8 and 10% and whose diameters were found between 110 and 360 nm, respectively. 

**Figure 2:**
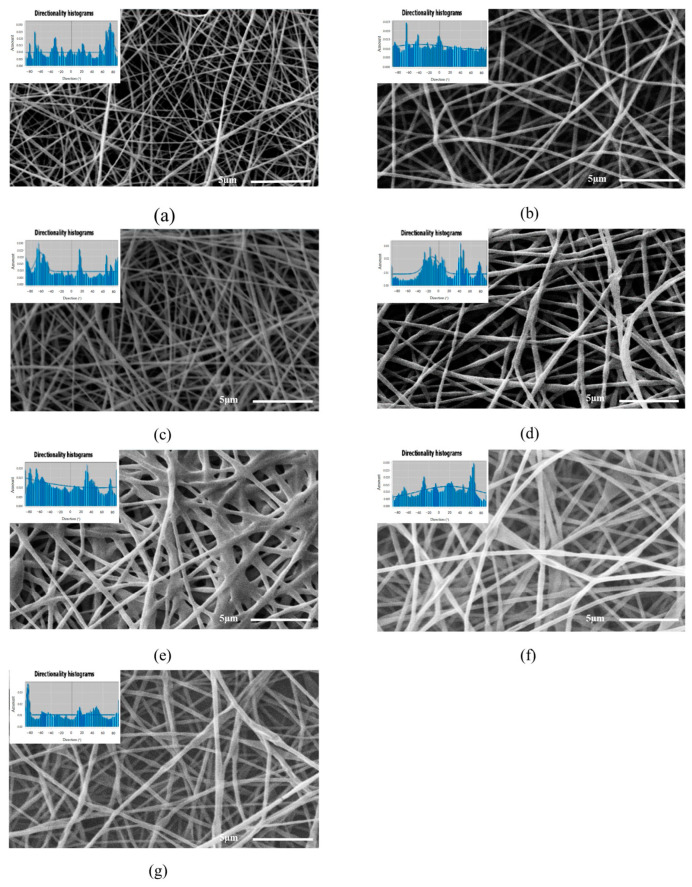
Micrographs of the employed systems: (**a**) PVOH 8% (**b**) PVOH 10% (**c**) PVOH 10%/OO 4%, (**d**) PVOH 10%/OO 8%, (**e**) PVOH 10%/OO 12%, (**f**) PVOH 10%/OEO 5% and (**g**) PVOH 10%/OEO 7.5%.

The SEM images of the electrospun nanofibers shown in Figure 2 indicate that the PVOH solutions at 8 and 10% *w*/*w*, Figure 2a,b, formed smooth nanofibers with homogeneous diameters, because their polymeric structure facilitated the stretching of the nanofiber during the electrospinning process [32,33], whereas the incorporation of vegetal oil during the formation of the nanofibers, Figure 2c–g, produced heterogeneous diameters as a result of the encapsulation of the vegetal oil in the nanofiber body. The mean diameters of the PVOH-OO and PVOH-OEO nanofibers oscillated between 208–492 nm and 266–300 nm, severally. With the increasing oil concentration, the viscosity of the emulsions grew and with it, the diameter of the electrospun nanofibers [34].

As for the moisture content, in Table 1, it is observed that it diminished as the PVOH concentration increased from 8 to 10% *w*/*w*; similar results occurred by increasing the oil concentration in the PVOH-OO and PVOH-OEO emulsions [35].

The diameters of the nanofibers displayed a wide distribution due to the electrospinning process and the different variables that affect it. These results are in good agreement with those reported by Kalantary et al. [36] who stated that the major contribution to the nanofiber diameters came from the polymer concentration in the solution to be electrospun. Xu et al. [37] reported that the viscosity increase was reflected in higher nanofiber diameters; on the other hand, Khajavi et al. [38] asserted that the conductivity diminution increased the nanofiber diameters. 

As for the orientation of the nanofibers in the studied systems, the analysis of the SEM images in Figure 2 by means of the software Orientation J 1.51j8 showed that the nanofibers are oriented randomly at different angles throughout the sample. Furthermore, it is observed that the nanofibers of the analyzed systems possess anisotropy, for the micrographs display multimodal histograms. This result was predictable, because the random orientation occurs when a spinning collector with low rotation rate of 150 rpm is used. Similar results were reported by Nitti et al. [39] who concluded that the angular velocity of the spinning drum affects significantly the orientation of the nanofibers and their anisotropic mechanical properties [40,41].

### 3.2. Determination of the Digital Porosity (DP)

The results corresponding to the DP calculation of the electrospun nanofibers from the SEM micrographs in Figure 1 are shown in Table 2.

The DP of the surface layers employing threshold I presented an average value of 40.12 ± 3.30, which is higher than the DP of intermediate layers obtained with threshold II with an average value of 27.44 ± 1.40, and finally, the DP of the intermediate and internal layers calculated with threshold III, which is lower than the previous ones with an average value of 19.66 ± 1.13, which was due to the fact that more nanofibers overlap and for this reason, the empty areas have lower magnitude. 

The physical characterization of the electrospun PVOH veils shows that the concentration and viscosity of the solution are important variables for controlling the nanofiber porosity. Notwithstanding, other factors can also affect the morphology of the electrospun material like the solution feeding rate, the injector-collector distance, voltage, etc. [42].

### 3.3. Proposed Model for Predicting the DP

With the analysis of the SEM micrographs and the image analysis technique, the development of a new mathematical expression was proposed to estimate the DP with experimental data obtained by image analysis. The preceded DP prediction model (${DP}_{P})$ is shown in Equation (7):

(7) $${DP}_{P}={\left(\frac{-\ln\left(\frac{V\times k\times d}{I}\right)(\ln(\frac{W_{f}}{W_{i}})}{e^{\theta }}\right)}^{n}$$

where V is the voltage (V), k is the conductivity (S/m), *W_f_* and *W_i_* are the final and initial moisture contents of the veil (g H_2_O)/(g b. s.), severally, *θ* is the electrospinning time (h), I is the electric current (A), d is the injector-collector distance (m) and n is a fractal exponent.

The mathematical expression was established according to the following criteria:

- In the literature, it has been reported that the morphology and properties of the nanofibers, including the diameter, porosity, alignment and mechanical behavior, depend on the polymer solution properties (concentration, viscosity, surface tension and dielectric properties); on processing parameters (voltage, volumetric flow rate, injector-collector distance and intensity of the applied electric field); and environmental conditions (temperature, atmospheric pressure and moisture) [43,44].
- Additionally, the electrospinning time was considered, because it is an important variable during the electrospinning process. Essaldi et al. [45] reported that the mean size of the space between nanofibers was smaller for longer electrospinning times.
- Finally, the final moisture content of the veil was taken into account, because during the electrospinning process, solution dehydration occurs as a consequence of solvent volatilization [46].

It is observed that Equation (7) is a function of the operation and solution parameters and moisture of the electrospun veils. Table 3 shows the values used in Equation (3) to predict the DP mathematically. 

**Figure 3:**
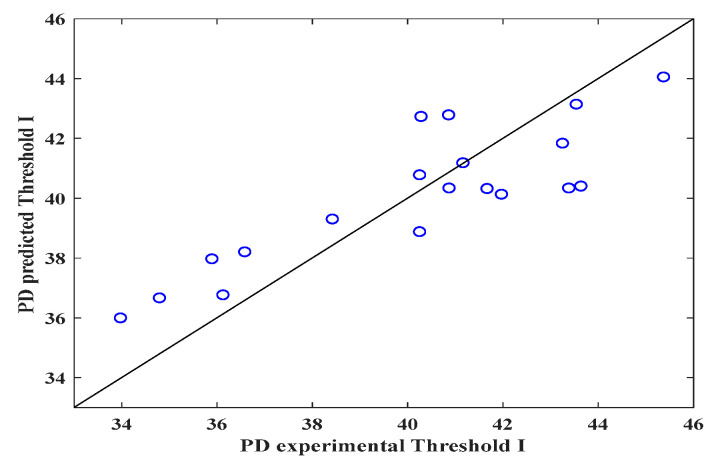
Predicted and experimental DP values for the electrospun veils, Threshold I.

**Figure 4:**
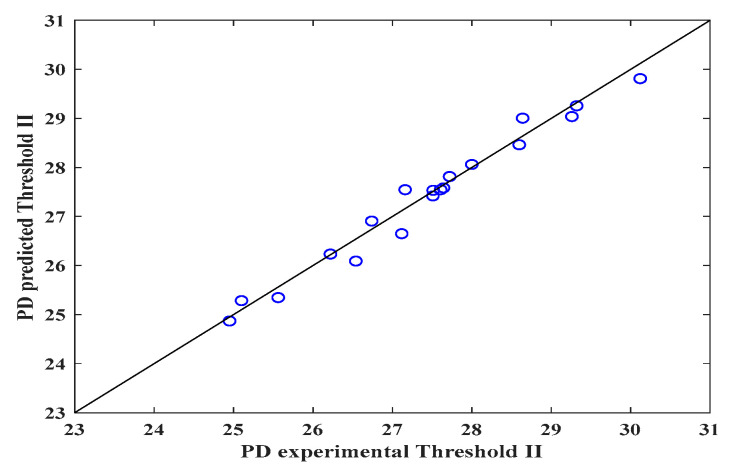
Predicted and experimental DP values for the electrospun veils, Threshold II.

**Figure 5:**
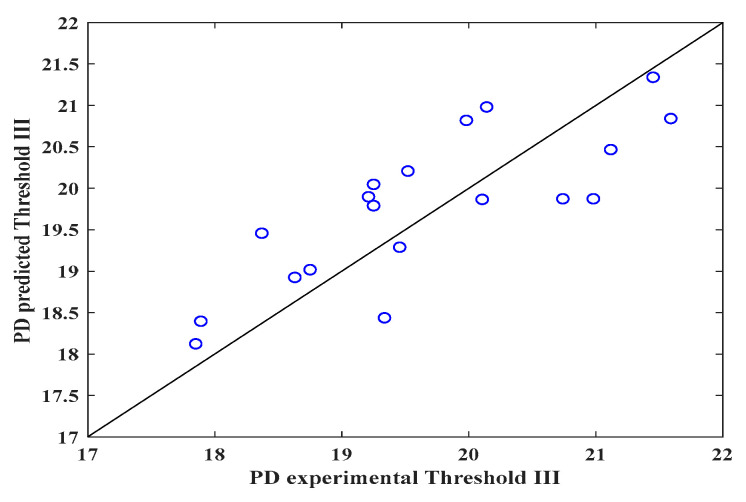
Predicted and experimental DP values for the electrospun veils, Threshold III.

Figure 3, Figure 4 and Figure 5 show the validity of the mathematical expression proposed to predict the DP. As can be observed in Figure 3 (threshold I), through the image analysis of the electrospun veils, there is a relationship between the calculated pore size and the DP calculated with the mathematical equation, obtaining R^2^ = 0.71, which indicates that the equation can predict the pore sizes in the surface layer of the electrospun veil; the RSS and MSE values featured in Table 4 represent the highest values with respect to those obtained with thresholds II and III, which reveals a certain error margin in the estimation of the DP. 

Conversely, Figure 4 shows that the mathematical expression improved the prediction (R^2^ = 0.97) with threshold II when the surface and intermediate layers of the electrospun veil SEM micrograph were employed, presenting the lowest RSS and MSE values, as observed in Table 4. Finally, in Figure 5, when threshold III was employed, once again the prediction diminished, due to the fact that the nanofibers overlapped, and the DP was reduced by the color contrasts. 

It is worth emphasizing that, for each threshold, the fractal exponent (*n*) of the mathematical expression is different. The statistical parameters (R^2^, R, RSS and MSE) for establishing the correlation between the experimental data and those predicted by the proposed equation are displayed in Table 4.

The statistical parameters for threshold II indicate that the equation correctly fits the calculated values, giving a prediction error of approximately 3%, whereas the mathematical expression for thresholds I and III had a prediction percentage error of 32 ± 3%. In a similar analysis, Powell et al. [47] proposed a correlation between the nanofiber porosity and composition of the solution of gelatin electrospun nanofibers, obtaining R^2^ = 0.70. Likewise, by means of response surface studies and RNA for porosity prediction of electrospun nanofibers, R^2^ = 0.94 and 0.89 were calculated, severally [43]. Furthermore, the physical characterization of the electrospun PVOH veils shows that the concentration and viscosity of the solution are fundamental variables for controlling the nanofiber porosity. Other factors, such as solution feeding rate, injector-collector distance and voltage, can affect the morphology of the electrospun veil [48]. Additionally, the voltage and conductivity affect the porosity of the electrospun nanofibers, for they are variables that are involved in the equation; however, the final moisture of the nanofibers is the most important factor because of the fact that, during electrospinning, a solution dehydration process occurs as a consequence of the volatilization of the solvents. The electrospinning time has an inversely proportional relationship with the DP. This is because the DP diminishes as the electrospinning time increases, which is a consequence of higher overlapping of the veil nanofibers. 

## 4. Conclusions

The image analysis method is a feasible option for establishing the surface DP of SEM micrographs of electrospun nanofiber veils. However, the method accuracy can be affected if a suitable selection of the threshold is not done; such an effect was observed when thresholds I and III were employed with R^2^ = 0.71 and 0.64, respectively, which indicated that the DP calculated with the equation can produce a certain error margin. 

Furthermore, the mathematical expression resulted highly reliable with threshold II, when surface and intermediate layers in the SEM micrographs of the electrospun veils were considered, yielding R^2^ = 0.97. For this reason, it is necessary that the right threshold be identified to reproduce as close as possible the pore areas during the electrospinning process of emulsions. 

With the proposed mathematical model, it was found that one of the important parameters to be considered is the moisture of the electrospun veils, for a dehydration process occurs during the electrospinning as a consequence of the volatilization of solvents, and another relevant parameter was the time.

## Figures and Tables

**Table 1:** Composition, moisture content, viscosity and conductivity of the PVOH-oil emulsions.

Sample Veil	Composition (% *w*/*w*)			Moisture Content (g.d.b)	Viscosity (Pa·s)	Conductivity (mS cm^−1^)	Nanofiber Diameter (nm)
	PVOH	Water	Oil				
1	8.00	92.00		11.50 ± 0.02	0.28 ± 0.02	0.58 ± 0.01	175.68 ± 4.11
2	10.00	90.00		9.00 ± 0.02	0.49 ± 0.02	0.48 ± 0.01	300.26 ± 7.97
With olive oil							
3	9.60	86.40	4.00	6.35 ± 0.02	0.39 ± 0.01	0.55 ± 0.01	211.57 ± 5.17
4	9.20	82.80	8.00	5.16 ± 0.02	0.43 ± 0.01	0.52 ± 0.01	293.81 ± 5.74
5	8.80	79.20	12.00	3.80 ± 0.02	0.46 ± 0.01	0.49 ± 0.01	485.19 ± 9.30
With orange essential oil							
6	9.50	85.50	5.00	5.89 ± 0.02	0.62 ± 0.01	0.45 ± 0.01	268.90 ± 2.09
7	9.25	83.25	7.50	4.97 ± 0.02	0.71 ± 0.01	0.43 ± 0.01	279.35 ± 18.41

**Table 2:** DP values obtained from SEM micrographs of electrospun nanofibers using different thresholds.

Sample Veil	Experimental Porosity (%)		
	Threshold I	Threshold II	Threshold III
1	43.12 ± 3.19	29.69 ± 0.61	21.52 ± 0.10
2	39.57 ± 2.04	27.65 ± 1.03	19.38 ± 0.51
3	40.47 ± 4.04	27.79 ± 1.41	19.34 ± 0.76
4	39.65 ± 1.73	26.95 ± 0.30	19.68 ± 1.85
5	34.38 ± 0.58	25.03 ± 0.11	17.87 ± 0.03
6	40.35 ± 3.74	27.22 ± 1.54	20.19 ± 0.89
7	42.42 ± 1.89	27.46 ± 0.29	19.80 ± 0.82

**Table 3:** Experimental data at 24 kV, distance of 0.2 m and electric current of 0.001 A is necessary to feed the empirical model.

Sample Veil	Conductivity K (S/m)	Moisture Polymeric Solution W_i_	Electrospun Veil W_f_	Electrospinning Time (Min)
1	0.058 ± 0.001	11.50 ± 0.02	0.02 ± 0.002	60.00 ± 1.00
2	0.048 ± 0.001	9.00 ± 0.02	0.01 ± 0.001	62.25 ± 2.06
3	0.055 ± 0.001	6.35 ± 0.02	0.01 ± 0.001	61.67 ± 3.51
4	0.052 ± 0.001	5.16 ± 0.02	0.01 ± 0.001	60.00 ± 1.00
5	0.049 ± 0.001	3.80 ± 0.02	0.01 ± 0.001	61.50 ± 0.71
6	0.045 ± 0.001	5.89 ± 0.02	0.01 ± 0.001	61.00 ± 3.61
7	0.043 ± 0.001	4.97 ± 0.02	0.01 ± 0.001	58.67 ± 1.15

**Table 4:** Statistical criteria for establishing the quality of the empirical fitting model.

Threshold	Fractal Exponent	R^2^	R	RSS	RMSE
I	1.10	0.71	0.84	56.70	1.68
II	0.99	0.97	0.98	1.01	0.22
III	0.89	0.64	0.80	8.82	0.66

## Data Availability

The data presented in this study are available on request from the corresponding author.
